# Comparing maximum diameter and volume when assessing the growth of small abdominal aortic aneurysms using longitudinal CTA data: cohort study

**DOI:** 10.1097/JS9.0000000000000433

**Published:** 2023-07-03

**Authors:** Robin Ristl, Johannes Klopf, Andreas Scheuba, Anna Sotir, Florian Wolf, Christoph M Domenig, Anders Wanhainen, Christoph Neumayer, Martin Posch, Christine Brostjan, Wolf Eilenberg

**Affiliations:** a Center for Medical Statistics, Informatics, and Intelligent Systems; b Department of General Surgery, Division of Vascular Surgery; c Department of Biomedical Imaging and Image Guided Therapy, Division of Cardiovascular and Interventional Radiology, Medical University of Vienna, Austria; d Department of Surgical Sciences, Uppsala University, Uppsala; e Department of Surgical and Perioperative Sciences, Surgery, Umeå University, Umeå, Sweden

**Keywords:** abdominal aortic aneurysm, aneurysm volume, computed tomography angiography, growth model, maximum aortic diameter

## Abstract

**Background:**

Monitoring of abdominal aortic aneurysms (AAAs) is currently based on serial measurements of maximum aortic diameter. Additional assessment of aneurysm volume has previously been proposed to possibly improve growth prediction and treatment decisions. To evaluate the use of supplementing volume measurements, the authors aimed to characterise the growth distribution of AAA volume and to compare the growth rates of the maximum diameter and volume at the patient level.

**Methods:**

Maximum diameter and volume were monitored every 6 months in 84 patients with small AAAs, with a total of 331 computed tomographic angiographies (with initial maximum diameters of 30–68 mm). A previously developed statistical growth model for AAAs was applied to assess the growth distribution of volume and to compare individual growth rates for volume and for maximum diameter.

**Results:**

The median (25–75% quantile) expansion in volume was 13.4 (6.5–24.7) % per year. Cube root transformed volume and maximum diameter showed a closely linear association with a within-subject correlation of 0.77. At the surgery threshold maximum diameter of 55 mm, the median (25–75% quantile) volume was 132 (103–167) ml. In 39% of subjects, growth rates for volume and maximum diameter were equivalent, in 33% growth was faster in volume and in 27% growth was faster in maximum diameter.

**Conclusion:**

At the population level, volume and maximum diameter show a substantial association such that the average volume is approximately proportional to the average maximum diameter raised to a power of three. At the individual level, however, in the majority of patient’s AAAs grow at different pace in different dimensions. Hence, closer monitoring of aneurysms with sub-critical diameter but suspicious morphology may benefit from complementing maximum diameter by volume or related measurements.

## Introduction

Highlights

- The growth of abdominal aortic aneurysms was analyzed based on longitudinal computed tomography scans and applying a stochastic growth model.
- In close over one half of patients, abdominal aortic aneurysms grow at different pace in different dimensions.
- At the surgery threshold of a maximum diameter of 55 mm, there is a large variability of corresponding aneurysm volume.
- Results suggest a relevant information gain through complementing maximum diameter measurements by volume or related measures.

Most abdominal aortic aneurysms (AAAs) that are detected through screening programs or as incidental findings are at a small size that does not indicate immediate surgical intervention. Small AAAs are monitored for growth and surgical treatment is offered depending on the assumed risk for rupture.

AAA maximum diameter has been established as the main parameter to quantify aneurysm growth and assess the requirement for surgical intervention, since it is a strong predictor for the risk of rupture^1^ and is easily measured using ultrasound scans. With the increasing use of computed tomography angiography (CTA), precise three-dimensional representations of AAAs may be obtained^2^ and, consequently, other parameters of AAA geometry have been discussed to assess changes in the size and shape of AAAs. Hatakeyama *et al.* ^3^ identified maximum diameter, the ratio of diameter, and length and diastolic blood pressure as relevant risk factors for rupture. Shum *et al.* ^4^ found that, among further related parameters, maximum diameter, volume, sac length, surface area, and intraluminal thrombus volume were predictive for rupture. Chandrashekaris *et al.* ^5^ found that the volume based undulation index and curvature were correlated with faster aneurysm growth. Similarly, Khan *et al.* ^6^ reported diameter, volume, and cross sectional area to be correlated with faster growth.

Kitagawa *et al.* ^7^ discussed that diameter may not fully address certain morphological variations such as saccular aneurysms, though they recommended the use of diameter and not volume due to the simpler measurement technique. Lindquist Liljeqvist *et al.* ^8^ concluded that volume has a stronger correlation with estimated biomechanical rupture risk compared to the diameter and they suggested to monitor AAAs in all three dimensions. Spanos *et al.* ^9^ reported for large AAAs an association between larger volume and increased risk for rupture.

Discordance between changes in diameter and in volume has been reported in previous AAA monitoring studies. Parr *et al.* observed patients with small AAAs over 14 months and reported an increase in volume (>6.08 ml) with stable orthogonal diameter (change ≤3.35 mm) in 19% of cases, stable volume and increasing diameter in 4%, and both parameters increasing in 23%^10,11^. Ghulam *et al.* ^11^ reported that among patients with small AAAs and stable 1-year growth (defined as +/-3.7 mm), 40% of patients showed considerable growth in volume (defined as >8.8 ml). In all three studies, margins for stable diameter or volume were based on the respective variability in repeated measurements. Therefore, the reported percentages of discordance depend on the precision of the measurement tools and a more detailed assessment of the relation of volume and diameter growth is still warranted.

In a recent publication^12^, a stochastic growth model has been developed for measurements of aneurysm size that takes into account known properties of AAA growth. Specifically, the model reflects that absolute growth depends on the current aneurysm size^13^, growth rates may vary within patients across time^14,15^, growth rates may vary between patients. The model allows for the estimation of, both, population parameters and subject specific growth rates^12^. Previously, the model was used to characterise the growth of AAA maximum diameter and to confirm a low probability for exceedance of different threshold diameters within recommended monitoring intervals.

Here, we address the question whether diameter and volume growth are typically in line with each other, or if and to what extent growth rates of these two parameters may deviate in the same subject. A close correlation in diameter and volume growth would imply that simple-to-obtain diameter measurements provide sufficient information regarding the overall extension of the aneurysm, and supplementing volume measurements would result in little information gain. In contrast, deviating growth patterns would strengthen the role of volume as an additional criterion in assessing the progression of AAAs.

We pursue three particular aims to characterise aneurysm growth in terms of volume and maximum diameter: (i) to give a comprehensive characterization of the natural growth of AAA volume by applying the stochastic growth model to volume data from precise longitudinally obtained CTA measurements, (ii) to assess the distribution of AAA volume at given maximum diameters, in particular at the threshold diameter of 55 mm, and (iii) to compare the individual growth rates for volume and maximum diameter within subjects to assess the percentages of patients in whom volume growth exceeds, is equivalent to, or below maximum diameter growth. For aims (ii) and (iii) we take into consideration that diameter and volume are related by a geometric power law.

## Methods

### Study population

Patients with known or newly diagnosed AAA visiting the outpatient clinic at the tertiary university hospital between 2014 and 2019 were prospectively included.

Data collection was approved by the local Ethics committee with license number 1729/2014 adhering to the principles of the Declaration of Helsinki and STROBE and STROCCS^16^ guidelines and registered at clinicaltrials.gov (NCT03507413) and ID researchregistry5637. All patients gave informed consent before participation in the study. Patients with recent malignancy or chemotherapy (<1-year), autoimmune disease, and organ transplantation were excluded. The morphometric growth analysis was performed at 6-month intervals for up to 5 years. For CTA, a Somatom Flash or Somatom Force instrument (Siemens) was applied with tube voltage settings of 120 or 100 kV and a tube current of 120 ref mAs (collimation: 2×64×0.6 mm). Images reconstructed in 1 mm slices were used for multiplanar reconstructions. Leading-edge-to-leading-edge diameter in axial corrected matter was defined as the maximum diameter. AAA volume was calculated by Syngovia. Morphometric AAA analysis (maximum aortic diameter and volume) was performed by two independent experts blinded to the previous CT measurement data, with mean values used for growth modeling and an intraclass correlation of 0.999^12^. Three patients in whom AAA volume was not measured were excluded for the current analysis. Two further subjects in whom AAA volume was measured at only one visit had to be excluded when estimating growth rates.

### Statistical analysis

For a descriptive summary of patient characteristics, the mean and SD were calculated for metric variables, and absolute and relative frequencies were calculated for categorical variables. The stochastic growth model, which was developed by Ristl *et al.* 2021^12^ to address specific properties of AAA expansion, was applied to assess the growth distribution of AAA volume in the studied population. The model assumes that each subject has an individual average growth rate λ , such that the expected relative growth within a time interval of t years is $e^{\lambda t}$ and the expected percent increase is $100(1-e^{\lambda t})$ . It is further assumed that the actual growth rate within each small-time interval is subject to random variations to reflect phases of faster or slower growth within the same subject. Additionally, it is assumed that between subjects, the average growth rates are randomly distributed according to a log-normal distribution. This is a right skewed distribution that reflects the observation that most patients show slow to moderate average growth rates while a small fraction of patients exhibits considerably faster growth. Using the model, the 5, 10, 25, 50 (median), 75, 90, and 95% quantiles and the mean of the 1-year growth distribution of AAA volume in the studied population were calculated. Computational details are provided in the online supplementary material, Supplemental Digital Content 1, http://links.lww.com/JS9/A745.

### Model validation

Leave-one-out cross-validation was applied to assess the validity of the stochastic growth model similar as described in Ristl *et al.* 2021. For each patient, the expected distribution of the volume at the last observed time-point was predicted from the initial volume measurement, using a model that was fitted with the data from all other patients. For the 5, 10, 25, 50, 75, 90, and 95% quantile of the predicted distribution, the percentage of cases in which the observed final value was below the predicted quantile was determined. For a model that correctly predicts the growth distribution, these percentages should correspond to the nominal percentages up to sampling variation.

### Correlation between maximum diameter and volume

When comparing changes in volume and maximum diameter, the different geometric units of volume and diameter (i.e. mm^3^ vs. mm) need to be considered. For any three-dimensional body, the volume V and the diameter d along one dimension are related by the equation $V=s*d^{3}$ , where s is a constant factor that depends on the actual shape of the body. Hence, to allow for direct comparison of volume and diameter at the same measurement scale, a cube root transformation to volume data, that is, calculating $V^{1/3}$ , was applied.

To study the distribution of the volume V that corresponds to a given maximum diameter d , a random effects model with equation $V_{\mathrm{ij}}^{1/3}=(a+\alpha_{i})*d_{\mathrm{ij}}+\epsilon_{\mathrm{ij}}$ was fit. Here, subscripts i and j refer to the j th measurement in the i th patient, the random slope $\alpha_{i}$ and the independent error term $\epsilon_{\mathrm{ij}}$ are modeled as normally distributed random variables with mean 0. Using this model, the median as well as the 5, 25, 75, and 95% quantile for the volume at a given maximum diameter were calculated as $V={\left(\hat{a}+z*{\hat{\sigma }}_{a}\right)}^{3}*d^{3}$ , where z is the corresponding quantile from a standard normal distribution (e.g. z=1.64 for the 95% quantile), $\hat{a}$ is the estimate for a and ${\hat{\sigma }}_{a}$ is the estimate for the standard deviation of the random effect $\alpha_{i}$ . As an illustrative example for the shape of an aneurysm we consider an ellipsoid with all circular cross sections with maximum diameter d and height h . For this case, we calculated the ratio between maximum diameter and height according to the equation $d/h=d^{3}/V*\mathrm{pi}/6$ .

The within-subject correlation between the cube root of volume and the maximum diameter was calculated using the correlation coefficient due to Bland and Altman for correlations with repeated measurements^17^, which is defined as the square root of the explained variation of one variable (cube root of volume) on another variable (maximum diameter) when adjusting for subject as a fixed effect. The coefficient has a similar interpretation as the usual Pearson correlation coefficient.

### Growth rates of maximum diameter and volume

To compare growth rates of volume and maximum diameter within the same patient, stochastic growth models were separately fit to the cube root volume data and to the maximum diameter data. From these models, the maximum likelihood estimates for the individual average growth rates (as percent per year) were calculated for each patient. Computational details are described in the online supplementary material, Supplemental Digital Content 1, http://links.lww.com/JS9/A745. Patients were categorized into five classes according to the ratio between their individual growth rates for cube root volume and for maximum diameter growth rates. Ratios between 0.8 and 1.25 were regarded to indicate equivalent growth in both parameters, ratios between 0.5 and 0.8 or 1.25 and 2 indicated moderately faster growth in one parameter compared to the other, and ratios below 0.5 or above 2 indicated a considerable deviation in growth rates with growth in one parameter being at least twice as fast as in the other parameter. Individual growth rates were compared between fusiform, saccular, and other aneurysms by descriptive summary statistics. For further descriptive comparisons, absolute and relative changes from baseline were calculated for volume and maximum diameter for each visit of each patient. Scatter plots were used for graphical representations. As a supplementary analysis, growth rates were also calculated for the subgroups of men, women, current smokers, and past or never smokers. All statistical analyses were performed using R version 4.2^18^.

## Results

### Characteristics of the patient sample

The study data comprised 331 CTA measurements of 84 patients (11 women and 76 men), for whom AAA growth was monitored at regular intervals of ~6 months. The AAA volume at baseline was between 17 and 207 ml with a median of 81 ml and the maximum diameter at baseline was between 30 and 68 mm with a median of 47 mm. The minimum, median, and maximum number of visits with CTA scans were 1, 4, and 9, respectively. Patient characteristics are shown in Table 1.

**Table 1:** Patient characteristics.

	Number of missing observations	Mean ± SD or absolute (relative) frequency of *n*=84
*Demography*		
Age at first visit [years]	0	71.6±7.7
Male sex	0	73 (87%)
BMI [kg/m^2^]	0	28.1±4.4
Follow-up time [years]	0	1.8±1.2
*Vascular characteristics*		
Maximum AAA diameter [mm] at first visit	0	46.3±7.6
AAA volume [ml] at first visit	0	85.2±46.2
Maximum thrombus [mm] at first visit	3	12.5±7.6
Thrombus volume [ml] at first visit	1	33.5±29.6
*Aneurysm morphology*		
Fusiform	0	63 (75%)
Saccular	0	16 (19%)
Eccentric	0	1 (1%)
Other	0	2 (2%)
Unknown	0	2 (2%)
*Smoking habits*		
Never smoker	0	5 (6%)
Past smoker	0	49 (58%)
Current smoker	0	30 (36%)
Packyears (of past and current smokers)	5	46.7±30.2
*Comorbidities*		
Hypertension	0	71 (85%)
Hyperlipidemia	0	70 (83%)
Peripheral artery disease	0	14 (17%)
Coronary heart disease	0	29 (34%)
Myocardial infarction	0	19 (23%)
Stroke	0	5 (6%)
Diabetes mellitus	0	19 (23%)
COPD	0	29 (34%)
*Medication*		
Antiplatelet therapy	0	75 (89%)
Anticoagulation therapy	0	16 (19%)
Antihypertensive therapy	0	71 (85%)
Lipid-lowering agents	0	79 (94%)
Diabetic medication	0	20 (24%)

Hypertension, hyperlipidaemia, peripheral artery disease, myocardial infarction, stroke, coronary heart disease, Diabetes mellitus and COPD (chronic obstructive pulmonary disease) were defined according to the AHA classification.

### Model validation

**Figure 1:**
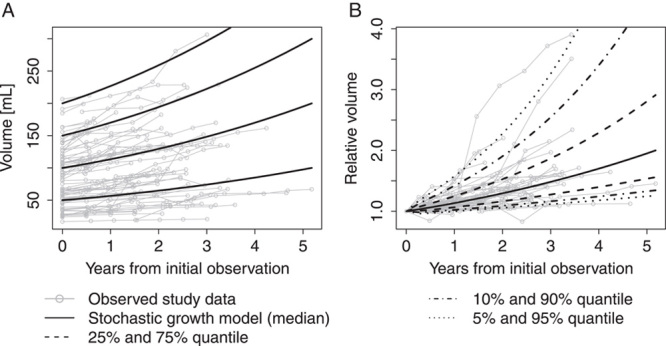
Absolute growth of abdominal aortic aneurysms volume (A). Gray lines indicate observed values in individual patients. Black lines show median trajectories as estimated from the stochastic growth model for particular starting values of 50, 100, and 150 ml volume. (B) Relative growth of abdominal aortic aneurysms volume and its growth distribution modeled by the stochastic growth model. Gray lines show the observed relative growth for each patient, which is defined as ratio of current value of maximum diameter and the value at the initial visit. Black lines represent estimates of selected quantiles of the relative growth distribution as estimated from the stochastic growth model.

The leave-one-out cross validation showed good agreement of the predictions of the stochastic growth model with the observed volume data for all considered quantiles, see Table 2. Similar to the previously reported model for the maximum diameter, the growth model for AAA volume is found to well describe the observed data and to address the skewed data distribution (Fig. 1).

**Table 2:** Leave-one-out cross validation. The table shows the percentages of patients for whom the observed final AAA volume was below the 5, 10, 25, 50, 75, 90, or 95% quantile that was predicted individually for each patient using the stochastic growth model. Standard errors (SE) for the percentages were calculated under the assumption of a perfect prediction model and are shown to assess the expected impact of sampling variability on deviations from the nominal quantile level.

Quantile level (%)	Percent below predicted quantile	SE
5	4.8	2.4
10	10.7	3.3
25	21.4	4.7
50	52.4	5.5
75	79.8	4.7
90	92.9	3.3
95	96.4	2.4

### Growth of AAA volume

Using the stochastic growth model, the mean growth rate of AAA volume was estimated at 19.3% per year, the estimated median was 13.4% per year and the interquartile range was 6.5–24.7%. In absolute terms this translates, for example, to a median 1-year growth of 7 ml, 13 ml, or 20 ml for aneurysms with a current volume of 50 ml, 100 ml, or 150 ml, respectively. Detailed statistics of the estimated growth distribution are shown in Table 3. The estimated time-trajectories for median growth and for selected quantiles for the relative growth distribution in the studied patient population are shown in Figure 1, together with the observed volume data. The nonlinearity of AAA growth is more pronounced for volume than for the maximum diameter and this aspect is reflected in the model-based reference curves. Estimates for model parameters are shown in the online supplementary material Supplementary Table S1, Supplemental Digital Content 1, http://links.lww.com/JS9/A745. An online tool to calculate quantiles of the volume growth distribution, as in Table 3. For research purposes was added to the calculator available at https://sny.cemsiis.meduniwien.ac.at/~zrx5rdf/jhG93c/.

**Table 3:** Distribution of 1-year growth of AAA volume estimated from the stochastic growth model. Mean and median increase as well as selected quantiles of the growth distribution are shown for a starting value of 100% (i.e. showing relative growth in percent) and for starting values of 50, 100, and 150 ml (i.e. showing absolute growth in ml). Standard errors (SE) are presented in parentheses below each value.

			Quantiles of 1-year growth distribution						
Growth	Initial value	Mean increase	5%	10%	25%	Median	75%	90%	95%
Relative (%)	100	19.3	−0.2	2.3	6.5	13.4	24.7	41.8	57.5
(SE)		(1.9)	(1.3)	(0.9)	(0.8)	(1.1)	(2.0)	(4.5)	(7.5)
Absolute (ml)	50	9.7	−0.1	1.1	3.3	6.7	12.3	20.9	28.8
(SE)		(1.0)	(0.7)	(0.5)	(0.4)	(0.6)	(1.0)	(2.2)	(3.8)
Absolute (ml)	100	19.3	−0.2	2.3	6.5	13.4	24.7	41.8	57.5
(SE)		(1.9)	(1.3)	(0.9)	(0.8)	(1.1)	(2.0)	(4.5)	(7.5)
Absolute (ml)	150	29.0	−0.3	3.4	9.8	20.1	37.0	62.7	86.3
(SE)		(2.9)	(2.0)	(1.4)	(1.2)	(1.7)	(3.1)	(6.7)	(11.3)

### Correlation between maximum diameter and volume

**Figure 2:**
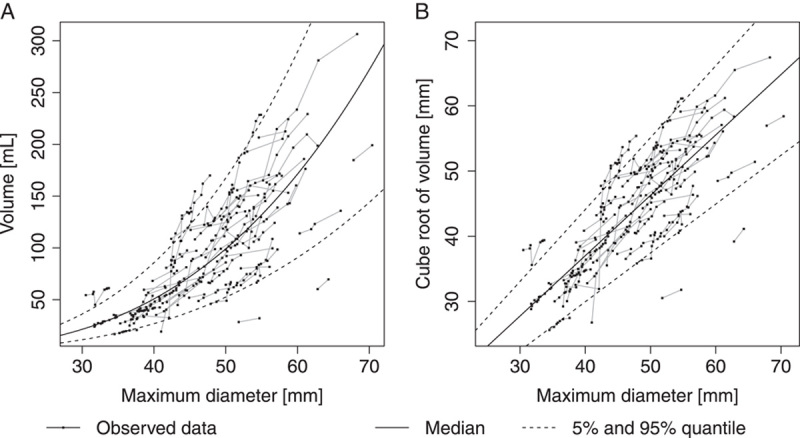
(A) Scatter plot of abdominal aortic aneurysms volume versus maximum diameter, and (B) Scatter plot of cube root transformed volume versus maximum diameter. Black dots connected by gray lines represent longitudinal measurements of the same patient. The solid black line represents the estimated median and dashed black lines indicate the estimated 5 and 95% quantile of the volume distribution given the maximum diameter.

The distribution of AAA volume for a given maximum diameter was calculated from the mixed model for cube root volume on maximum diameter as detailed in the methods section. Results are summarized in Table 4. The estimated volume distribution for a maximum diameter at the surgery threshold of 55 mm had a median of 132 ml and the 5 and95% quantiles were 70 ml and 223 ml. The wide range of the volume distribution indicates that among patients with the same maximum diameter the aneurysm volumes may vary considerably. For illustration, for an aneurysm of ellipsoid shape with circular cross sections, the volume distribution at a given maximum diameter would translate into a ratio between maximum diameter and height with a median of 0.66 and a 5–95% quantile range of 1.25–0.39. The cube root of volume and the maximum diameter showed a closely linear relationship, both, across the studied population and within individual subjects (Fig. 2). Linearity within a subject along a line with intercept 0 (such as the reference lines in Fig. 2B) in particular is observed if an aneurysm grows equally in all three dimensions while the overall shape stays the same. At the initial visit, the Pearson correlation between cube root volume and maximum diameter was 0.74. The within-subject correlation for cube root volume and maximum diameter, taking-into account all longitudinal measurements, was 0.77. A strong association with maximum diameter is also observed for untransformed volume measurements with a correlation of 0.72 at the initial visit and a within-subject correlation of 0.74; however, due to the geometric power law between volume and diameter, the association at the original scale is nonlinear (Fig. 2A).

**Table 4:** Estimated distribution of AAA volume at selected maximum diameters.

	Quantiles of volume distribution				
Maximum diameter	5%	25%	Median	75%	95%
30	11.3	16.8	21.4	26.9	36.2
40	26.9	39.7	50.8	63.6	85.8
50	52.5	77.6	99.1	124.3	167.5
55	69.9	103.3	132.0	165.5	223.0
60	90.7	134.1	171.3	214.8	289.5

### Comparison of growth rates for maximum diameter and volume

**Figure 3:**
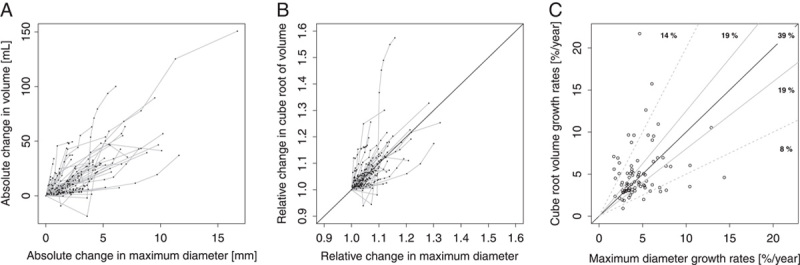
(A) Absolute change from baseline in abdominal aortic aneurysms volume versus absolute change from baseline in maximum diameter, and (B) Relative change from baseline in cube root of volume versus relative change from baseline in maximum diameter. Black dots connected by gray lines represent longitudinal measurements of the same patient. The diagonal reference line indicates identical relative growth in both parameters. (C) Scatter plot of estimated individual growth rates for volume and maximum diameter. The diagonal solid black line indicates identical relative growth in both parameters. Solid gray lines represent ratios between the two growth rates of 0.8 and 1.25, dashed gray lines represent ratios of 0.5 and 2.0. Inlayed numbers show the proportion of patients within the respective reference ratios.

The comparison of volume and maximum diameter growth in terms of absolute or relative changes from baseline is shown in Figures 3A and 3B. The comparison of individual growth rates and the categorization by the ratio of the two growth rates are shown in Figure 3C. In 39% (*n*=33) of all subjects, growth in volume, and in maximum diameter were at comparable rates, with the ratio between the estimated individual growth rates for cube root volume and maximum diameter within the equivalence margin of 0.8–1.25. In 19% (*n*=16) subjects, growth in volume was faster but less than twice as fast (at the cube root transformed scale) than the growth of maximum diameter. Another 19% (*n*=16) showed the opposite behavior, with growth in maximum diameter being faster but less than twice as fast than volume growth. In 14% (*n*=12) growth in volume was more than twice as fast as growth in maximum diameter, and in 8% (*n*=7) growth in maximum diameter was more than twice as fast as growth in volume. The graphical representations in Figure 3 and the comparison of growth rates indicate that in less than half of all patients, volume, and maximum diameter grow at comparable rates whereas in more than half of all patients, volume, and maximum diameter grow at different rates and that the number of patients with faster growth in volume and those with faster growth in maximum diameter are almost evenly balanced.

A similar pattern was observed when separately analyzing the subgroups of men, women, current smokers, and past or never smokers (Supplementary Figure S1, Supplemental Digital Content 1, http://links.lww.com/JS9/A745). In women, the observed growth in diameter was slightly stronger than the growth in volume; however, the differences of growth rates between men and women were within possible sampling variability. Smoker status did not affect growth rates. See Supplementary Table S2, Supplemental Digital Content 1, http://links.lww.com/JS9/A745 for estimated growth rates within subgroups.

A descriptive comparison of the estimated individual growth rates between different morphologies shows ~20% faster growth of, both, maximum diameter and cube root volume in saccular compared to fusiform aneurysms (Table 5 and Supplementary Figure S3, Supplemental Digital Content 1, http://links.lww.com/JS9/A745). The ratio between growth rates for cube root volume and maximum diameter was similar across major morphologies with a median ratio of 1.0 in fusiform and 1.09 in saccular aneurysms, see Table 5 and Supplementary Figures S2 and S3, Supplemental Digital Content 1, http://links.lww.com/JS9/A745.

**Table 5:** Descriptive summary of individual growth rates by aneurysm morphology. The table shows median (minimum–maximum) of the estimated individual growth rates in percent, as well as the ratio between growth rates for cube root volume and maximum diameter, *n* refers to the number of observed aneurysms by morphology.

		Median (minimum–maximum) of 1-year growth rates [%]			
Morphology	*n*	Diameter	Cube root volume	Volume	Cube root volume/diameter
Fusiform	63	3.8 (1.7−12.9)	3.8 (0.9−15.7)	11.7 (2.8−55)	1.00 (0.34−4.19)
Saccular	16	4.6 (3.1−14.4)	4.7 (3−21.7)	14.7 (9.1−80.3)	1.09 (0.32−4.65)
Eccentric	1	6.8 (6.8−6.8)	10.9 (10.9−10.9)	36.6 (36.6−36.6)	1.61 (1.61−1.61)
Other	2	4.6 (3.2−5.9)	4 (3−5.1)	12.6 (9.2−16.1)	1.04 (0.51−1.57)
Unknown	2	2.7 (1.9−3.5)	3.8 (3.1−4.5)	11.8 (9.4−14.1)	1.64 (0.88−2.41)

## Discussion

The decision to include volume data in regular monitoring of AAA growth comes at the cost of greater technical efforts and, when using precise CTA, increased radiation exposure for patients compared to ultrasound examinations. We aimed to further advance the assessment of volume data by providing a comprehensive description of the natural growth distribution of volume, via a novel growth model for volume expansion, the association of volume and maximum diameter, and the association of their growth rates.

When comparing the growth of volume and diameter, the power law in the geometric relation of volume and diameter needs to be taken-into account, as detailed in the Methods section. Indeed, the distribution of growth rates for cube root transformed volume and for maximum diameter was found to be similar. This indicates that at the population level and accounting for the different geometric units, the growth of volume and diameter is approximately at the same rate. At the individual level; however, there is considerable heterogeneity. Previous studies reported a considerable fraction of patients in whom aneurysm growth greater than a threshold was observed in one parameter only^10,11^. By a direct comparison of growth rates, brought to the same measurement scale using the cube root transformation, we found that only in 39% of subjects, growth rates for volume and maximum diameter were equivalent, whereas in 33% growth was faster in volume and in 27% growth was faster in maximum diameter. This indicates that in a majority of patients, AAAs do not grow equally in all dimensions and that the proportions of patients with excess growth in either parameter are approximately balanced.

In patients in whom aneurysm growth is stronger in a dimension other than the maximum diameter (e.g. if height or terminal diameters increase stronger), a focus on the maximum diameter may not reflect the actual disease progression and may be prone to overlooking morphological changes relevant to the risk of rupture. It is known that patients with a similar AAA growth in maximum aortic diameter do not necessarily have the same risk for rupture, even when exceeding the 55 mm threshold for surgical repair^19^. Volume measurements capture the overall extension, which should be a particularly sensitive measure to complement information from the maximum diameter.

Saccular and fusiform AAAs showed similar behavior in terms of growth in volume and maximum diameter, and in particular the heterogeneity in volume versus diameter growth rates was similar for both morphologies. Hence, for both morphologies, monitoring AAA growth in different dimensions may aid to detect a relevant progression in time and a strong growth in volume between two visits may in general be considered as an important signal that warrants closer monitoring even if the maximum diameter is below a critical value. However, saccular AAAs are at increased risk of rupture even at smaller size, compared to fusiform AAAs^20,21^. Hence, additional measurements to detect relevant changes in time may of particular importance for small saccular AAAs.

According to current guidelines, in fusiform AAAs a maximum diameter below 40 mm indicates a low risk of rupture and ensues the recommendation for further monitoring, whereas a maximum diameter greater than or equal to 55 mm indicates the need for surgical repair^22,23^. Similar thresholds are missing for volume. Our results show that across patients with the same diameter, aneurysm volumes vary considerably, hence, a direct translation of diameter thresholds to volume does not seem feasible. Volume measurements would be obtained with largest precision using CT angiography; however, the possible information gain from CT needs to be weighed against individual disease state and radiation exposure. Of note, the benefit-risk ratio of CT angiography may in the future further improve as technological advances allow for machines with reduced radiation intensity.

Hence, complementation of maximum diameter measurement by CT based volume (or related measures) may be advisable in particular for larger or irregularly shaped aneurysms or aneurysms considered to be at increased risk of rupture such as saccular. In other cases, it may be sufficient to assess changes in aneurysm size and shape by high resolution 2D or 3D ultrasound^24^. In regularly shaped aneurysms, measuring the ratio of diameter and length may give approximately the same additional information as measuring volume, because for an ellipsoid with all circular cross sections, volume is a function of only height and diameter. Indeed, Hatakeyama *et al*.^3^ found that the combination of maximum diameter and the ratio of diameter and length improved the prediction of rupture compared to the predictive power of each parameter separately.

Parallel to our study, Olson *et al.* ^25^ investigated the growth of AAA volume by CT in a cohort of 250 patients in the Unites States with a maximum follow-up of 2 years. They found a growth rate median of 9.3 and a mean of 10.4%, which is less than the growth rate median of 13.4 and mean of 19.3% we observed. The larger difference for the mean growth rate, as opposed to the difference in median growth rates, may be attributed to the longer observation time frame in our study of up to 5 years. A longer follow-up increases the possibility to observe rare cases with phases of unusually fast growth, and the mean, but not the median, depends strongly on such extreme values. Olson *et al.* found a Pearson correlation of 0.66 between volume and maximum diameter at the first visit. We observed a larger value of 0.72; however, taking-into account the sample sizes, this difference is not statistically significant. Of note, due to the nonlinear relation of volume and diameter, the Pearson correlation will underestimate the extent of association, and accordingly we found an even larger correlation between cube root volume and maximum diameter. Olson *et al.* did not investigate the association of volume and diameter growth rates. Importantly, similar to our study they observed that the volume at the 55 mm threshold diameter shows high variation. Olson *et al.* inferred from the observed correlation of volume and diameter and the large variation of volume at the threshold diameter that volume should not play a major role in determining the need for surgery. However, one may alternatively argue that, if rupture risk also depends on AAA morphology other than maximum diameter, the large variability in volume may offer the potential to discriminate between patients with high and low risk of rupture below the current diameter threshold. In line with Olson *et al.*, we acknowledge that data relating volume to clinical outcomes is scarce, and large prospective studies will be required to confirm volume as clinical determinant of adverse outcome. As discussed, the ratio of length over diameter may serve as a proxy for volume and this type of data is likely to be more abundant than volume measurements in historic data sets. Therefore, retrospective analysis of length over diameter as predictor of rupture could be of value.

According to the concept of radiomics, future assessment of risk could also be based on high dimensional data and subsequent model selection^26^. In a recent feasibility study, Rezaeitaleshmahalleh *et al.* combined several geometric, hemodynamic and patient health features to predict fast versus slow growing AAAs, with geometry found to be a relevant predictor^27^. Future research may aim at extending guidelines for measures that complement maximum diameter.

Some limitations of the study must be acknowledged. Only 84 patients were included, which is a limited cohort. All patients were Caucasian from central Europe, larger groups with different ethnicities all around the world would increase the value of the study. All patients in our study had degenerative AAAs and extrapolations of our results to populations with different etiology, such as connective tissue disorder, should be made with caution. Strengths of the study are the longitudinal character with a large number of 331 repeated measurements in total and the high precision of obtained CTA images and derived diameter and volume data.

In conclusion, when deciding on a set of outcome variables for an epidemiologic study or a clinical trial, the additional gain through volume measurements is likely limited and maximum diameter measurements may be sufficient. At the individual subject level; however, closer monitoring of growth by including volume or related measures may provide information to detect risk relevant changes earlier than by monitoring only maximum diameter. Such an information would be of greatest relevance in AAAs at larger risk of rupture, as fusiform AAAs above, for example, a diameter of 40 mm and in saccular AAAs regardless of size.

## Ethical approval

The institutional ethics committee of the Medical University of Vienna approved the study in all details of the study protocol. (license no. 1729/2014 and 1479/2017).

## Sources of funding

This work was primarily supported by the Austrian Science Fund (SFB project F 5409-B21) as well as the Medical Scientific Fund of the Mayor of the City of Vienna (project 15012).

## Author contribution

R.R.: conceptualization, formal analysis, data curation, investigation, roles/writing, original draft, writing - review and editing; J.K.: methodology, roles/writing, writing - review and editing; A.S.: data curation, methodology, original draft, writing - review and editing; A.S.: methodology, writing - review and editing; F.W.: data curation, roles/writing, original draft, writing - review and editing; C.M.D.: conceptualization, roles/writing, original draft, writing - review and editing; A.W.: investigation, roles/writing, writing - review and editing; C.N.: formal analysis, investigation, roles/writing, original draft, writing - review and editing; M.P.: conceptualization, formal analysis, roles/writing, original draft, writing - review and editing; C.B.: conceptualization, formal analysis, roles/writing, original draft, writing - review and editing; W.E.: conceptualization, formal analysis, data curation, investigation, roles/writing, original draft, writing - review and editing. All authors are responsible for the conception and design of the study, acquisition of data, analysis and interpretation of data, drafting the article and revising it critically for important intellectual content and final approval of the version to submission.

## Conflicts of interest disclosure

The authors declare that they have no financial conflict of interest with regard to the content of this report.

## Research registration unique identifying number (UIN)

1. Registry used: https://www.researchregistry.com/.
2. Unique Identifying number or registration ID: ID researchregistry5637.
3. Hyperlink to your specific registration (must be publicly accessible, not open on the homepage or the search/database listing and will be checked).

## Guarantor

Wolf Eilenberg.

## Provenance and peer review

Not commissioned, externally peer-reviewed.

## Supplementary Material

## References

[1] Vardulaki KA Prevost TC Walker NM. Growth rates and risk of rupture of abdominal aortic aneurysms. Br J Surg 1998;85:1674–1680. 9876073 10.1046/j.1365-2168.1998.00946.x

[2] van Prehn J van der Wal MB Vincken K. Intra- and interobserver variability of aortic aneurysm volume measurement with fast CTA postprocessing software. J Endovasc Ther 15 United States 2008;15:504–510. 10.1583/08-2478.1 18840051

[3] Hatakeyama T Shigematsu H Muto T. Risk factors for rupture of abdominal aortic aneurysm based on three-dimensional study. J Vasc Surg 33 United States 2001;33:453–461. 10.1067/mva.2001.111731 11241112

[4] Shum J Martufi G Di Martino E. Quantitative assessment of abdominal aortic aneurysm geometry. Ann Biomed Eng 2011;39:277–286. 20890661 10.1007/s10439-010-0175-3 PMC3070409

[5] Chandrashekar A Handa A Lapolla P. Prediction of abdominal aortic aneurysm growth using geometric assessment of computerized tomography images acquired during the aneurysm surveillance period. Ann Surg 2023;277:e175–e83. 33630463 10.1097/SLA.0000000000004711 PMC8691375

[6] Khan M Rogers S Carreira J. Aneurysm Geometry Analyzed by the Novel Three-Dimensional Tomographic Ultrasound Relates to Abdominal Aortic Aneurysm Growth. Ann Vasc Surg 2022;87:469–477. 35667487 10.1016/j.avsg.2022.05.017

[7] Kitagawa A Mastracci TM von Allmen R. The role of diameter versus volume as the best prognostic measurement of abdominal aortic aneurysms. J Vasc Surg 2013;58:258–265. 23806259 10.1016/j.jvs.2013.05.001

[8] Lindquist Liljeqvist M Hultgren R Gasser TC. Volume growth of abdominal aortic aneurysms correlates with baseline volume and increasing finite element analysis-derived rupture risk. J Vasc Surg 2016;63:1434–42 e3. 27106248 10.1016/j.jvs.2015.11.051

[9] Spanos K Nana P Kouvelos G. Anatomical differences between intact and ruptured large abdominal aortic aneurysms. J Endovasc Ther 2020;27:117–23. 31709885 10.1177/1526602819886568

[10] Parr A Jayaratne C Buttner P. Comparison of volume and diameter measurement in assessing small abdominal aortic aneurysm expansion examined using computed tomographic angiography. Eur J Radiol 2011;79:42–47. 20061105 10.1016/j.ejrad.2009.12.018

[11] Ghulam QM Bredahl KK Lonn L. Follow-up on small abdominal aortic aneurysms using three dimensional ultrasound: volume versus diameter. Eur J Vasc Endovasc Surg 2017;54:439–45. 28765014 10.1016/j.ejvs.2017.06.016

[12] Ristl R Klopf J Scheuba A. Growth prediction model for abdominal aortic aneurysms. Br J Surg 2022;109:211–219. 34849588 10.1093/bjs/znab407 PMC10364708

[13] Isselbacher EM. Thoracic and abdominal aortic aneurysms. Circulation 2005;111:816–828. 15710776 10.1161/01.CIR.0000154569.08857.7A

[14] Brady AR Thompson SG Fowkes FG. Participants UKSAT. Abdominal aortic aneurysm expansion: risk factors and time intervals for surveillance. Circulation 2004;110:16–21. 15210603 10.1161/01.CIR.0000133279.07468.9F

[15] Bailey MA Baxter PD Jiang T. Modeling the growth of infrarenal abdominal aortic aneurysms. Aorta (Stamford) 2013;1:268–273. 26798704 10.12945/j.aorta.2013.13-036 PMC4682719

[16] Agha R Abdall-Razak A Crossley E. STROCSS 2019 guideline: strengthening the reporting of cohort studies in surgery. Int J Surg 2019;72:156–165. 31704426 10.1016/j.ijsu.2019.11.002

[17] Bland JM Altman DG. Calculating correlation coefficients with repeated observations: part 1–Correlation within subjects. BMJ 1995;310:446. 7873953 10.1136/bmj.310.6977.446 PMC2548822

[18] (2015) RCT. R: A language and environment for statistical computing. R Foundation for Statistical Computing, Vienna, Austria. 2015.

[19] Collaborators R Bown MJ Sweeting MJ. Surveillance intervals for small abdominal aortic aneurysms: a meta-analysis. JAMA 2013;309:806–813. 23443444 10.1001/jama.2013.950

[20] Karthaus EG Tong TML Vahl A. Saccular abdominal aortic aneurysms: patient characteristics, clinical presentation, treatment, and outcomes in the Netherlands. Ann Surg 2019;270:852–858. 31498185 10.1097/SLA.0000000000003529

[21] Kristmundsson T Dias N Resch T. Morphology of small abdominal aortic aneurysms should be considered before continued ultrasound surveillance. Ann Vasc Surg 2016;31:18–22. 26631772 10.1016/j.avsg.2015.09.016

[22] Chaikof EL Dalman RL Eskandari MK. The society for vascular surgery practice guidelines on the care of patients with an abdominal aortic aneurysm. J Vasc Surg 2018;67:2–77 e2. 29268916 10.1016/j.jvs.2017.10.044

[23] Wanhainen A Verzini F Van Herzeele I. Editor’s Choice - European Society for Vascular Surgery (ESVS) 2019 Clinical Practice Guidelines on the Management of Abdominal Aorto-iliac Artery Aneurysms. Eur J Vasc Endovasc Surg 2019;57:8–93. 30528142 10.1016/j.ejvs.2018.09.020

[24] Zielinski AH Bredahl KK Ghulam QM. Full-Volume Assessment of Abdominal Aortic Aneurysm by Improved-Field-of-View 3-D Ultrasound Performs Comparably to Computed Tomographic Angiography. Ultrasound Med Biol 2022;48:283–292. 34823944 10.1016/j.ultrasmedbio.2021.10.018

[25] Olson SL Panthofer AM Blackwelder W. Role of volume in small abdominal aortic aneurysm surveillance. J Vasc Surg 2022;75:1260–1267 e3. 34655683 10.1016/j.jvs.2021.09.046 PMC8940629

[26] Mayerhoefer ME Materka A Langs G. Introduction to radiomics. J Nucl Med 2020;61:488–95. 32060219 10.2967/jnumed.118.222893 PMC9374044

[27] Rezaeitaleshmahalleh M Sunderland KW Lyu Z. Computerized differentiation of growth status for abdominal aortic aneurysms: a feasibility study. J Cardiovasc Transl Res 2023. online ahead of print. 10.1007/s12265-022-10352-8 36602668

