# Supplementary material for: Comparing maximum diameter and volume when assessing the growth of small abdominal aortic aneurysms using longitudinal CTA data: cohort study
Source: Int J Surg. 2023 Jul 3;109(8):2249–57. doi: 10.1097/JS9.0000000000000433 (PMC10442135; doi:10.1097/JS9.0000000000000433)
Supplement: Supplementary file 1 [file js9-109-2249-s001.pdf]

Supplementary material for "Comparing maximum diameter and volume when assessing the growth of small abdominal aortic aneurysms using longitudinal CTA data"

1. Computational details for the stochastic growth model

Notation: For subjects $i = 1, \dots, n$ denote the vector of observed AAA size (e.g. maximum diameter or volume) as $(y_{i0}, y_{i1} \dots, y_{im_i})$ and denote with δ_j the time intervals between observations y_{ij} and $y_{i(j-1)}$. Let $r_{ij} = \log\left(\frac{y_{ij}}{y_{i(j-1)}}\right)$ and denote with $\varphi(.\,; \mu, \sigma^2)$ and $\Phi(.\,; \mu, \sigma^2)$ the density and the distribution function, respectively, of a normal distribution with mean μ and variance σ^2.

The stochastic growth model can be regarded as a geometric Brownian motion extended by a log-normally distributed random effect for the scale parameter.

The model equation is $\frac{Y_{ij}}{Y_{il}} = \exp\left\{\left(\lambda_i - \frac{\sigma^2}{2}\right)\delta_j + \sigma W_{\delta_j}\right\}$, where λ_i is the patient specific growth rate parameter, W_{δ_j} denotes the a Brownian motion process at time δ_j, and the parameter σ determines the within-patient variability of the growth rate. It is further assumed that the within-patient variability is proportional to the growth rate λ_i by a scale factor k, i.e. $\sigma = k * \lambda_i$.

To address between-patient heterogeneity in growth rates, a log-normally distributed random effect for λ_i is assumed with parameters mean θ and variance ν^2, i.e. $\log(\lambda_i) \sim N(\theta, \nu^2)$ independently for each subject.

Maximum likelihood estimation: The maximum likelihood estimators for θ, ν and k are found such that the likelihood $\prod_{i=1}^{n}\int_{-\infty}^{\infty}\prod_{j=1}^{m_i}\varphi\left(r_{ij};\left(e^{\beta} - \frac{1}{2}\left(ke^{\beta}\right)^2\right)\delta_{ij}, \left(ke^{\beta}\right)^2\delta_{ij}\right)\varphi(\beta;\theta,\nu^2)d\beta$ is maximised. To facilitate computations, a Laplace approximation to the likelihood was applied in the optimisation algorithm.

Probabilities, quantiles and mean for relative increase: The probability that, in a new subject with current AAA size y_t, the relative increase over a time interval δ is less or equal q is $P\left(\frac{Y_{t+\delta}}{Y_t} \leq q\right) = \int_{-\infty}^{\infty}\Phi\left(\log q\,; \left(e^{\beta} - \frac{1}{2}\left(ke^{\beta}\right)^2\right)\delta, \left(ke^{\beta}\right)^2\delta\right)\varphi(\beta;\theta,\nu^2)d\beta$. Quantiles were

calculated by numeric integration of this equation and numeric root finding to solve for q. The population mean is given by the integral $\int_{-\infty}^{\infty} \exp(\exp(\beta)\,\delta)\,\varphi(\beta;\theta,\nu^2)d\beta$, which was solved numerically. Parameters were replaced by their estimates in actual calculations of probabilities and quantiles.

Estimation of individual growth rate: Updated probabilities based on previous measurements: For a given patient denote the vector of previously observed AAA sizes as $(y_0, y_1 \dots, y_m)$ and denote with δ_j the time intervals between observations y_j and y_{j-1} and let $r_j = \log\left(\frac{y_j}{y_{j-1}}\right)$. Conditional on the known observations for patient i, the density of the random effect β for this patient is $f(\beta;\theta,\nu^2|y_{i0},\dots,y_{im},\delta_{i1},\dots,\delta_{im}) = \frac{g(\beta)}{\int_{-\infty}^{\infty} g(\beta)d\beta}$, with $g(\beta) = \prod_{j=1}^{m} \varphi\left(r_{ij};\left(e^{\beta} - \frac{1}{2}\left(ke^{\beta}\right)^2\right)\delta_{ij},\left(ke^{\beta}\right)^2\delta_{ij}\right)\varphi(\beta;\theta,\nu^2)$. Let $\hat{\beta}_i$ be maximizer of this conditional density for subject i. Then the maximum likelihood estimator for λ_i is $\hat{\lambda}_i = \exp(\hat{\beta}_i)$. Given the patient-specific growth parameter λ_i, the individual mean of the relative increase $\frac{Y_{i(t+\delta)}}{Y_{it}}$ over a time interval δ is $e^{\lambda_i\delta}$. With time units in years, the according estimate for the individual one-year relative growth rate is $\exp(\hat{\lambda}_i)$.

Actual calculations of $\hat{\lambda}_i$ were performed by numeric optimisation, and in the conditional density the maximum likelihood estimates for θ, ν and k were plugged in, such that the method can be regarded as empirical Bayesian estimation.

Programming code for the statistical programming language R to fit the stochastic growth model is available at https://github.com/rmaster1/SGM .

Estimated model parameters

Supplementary Table S1. Parameter estimates and their standard errors (SE) of the fitted stochastic growth model for AAA volume.

Parameter	Estimate	SE
Location parameter θ	-1.951	0.081
Between subject-variability parameter ν	0.598	0.057
Within subject-variability parameter k	0.600	0.041

2. Subgroup analyses for men, women, current smokers and past or never smoker

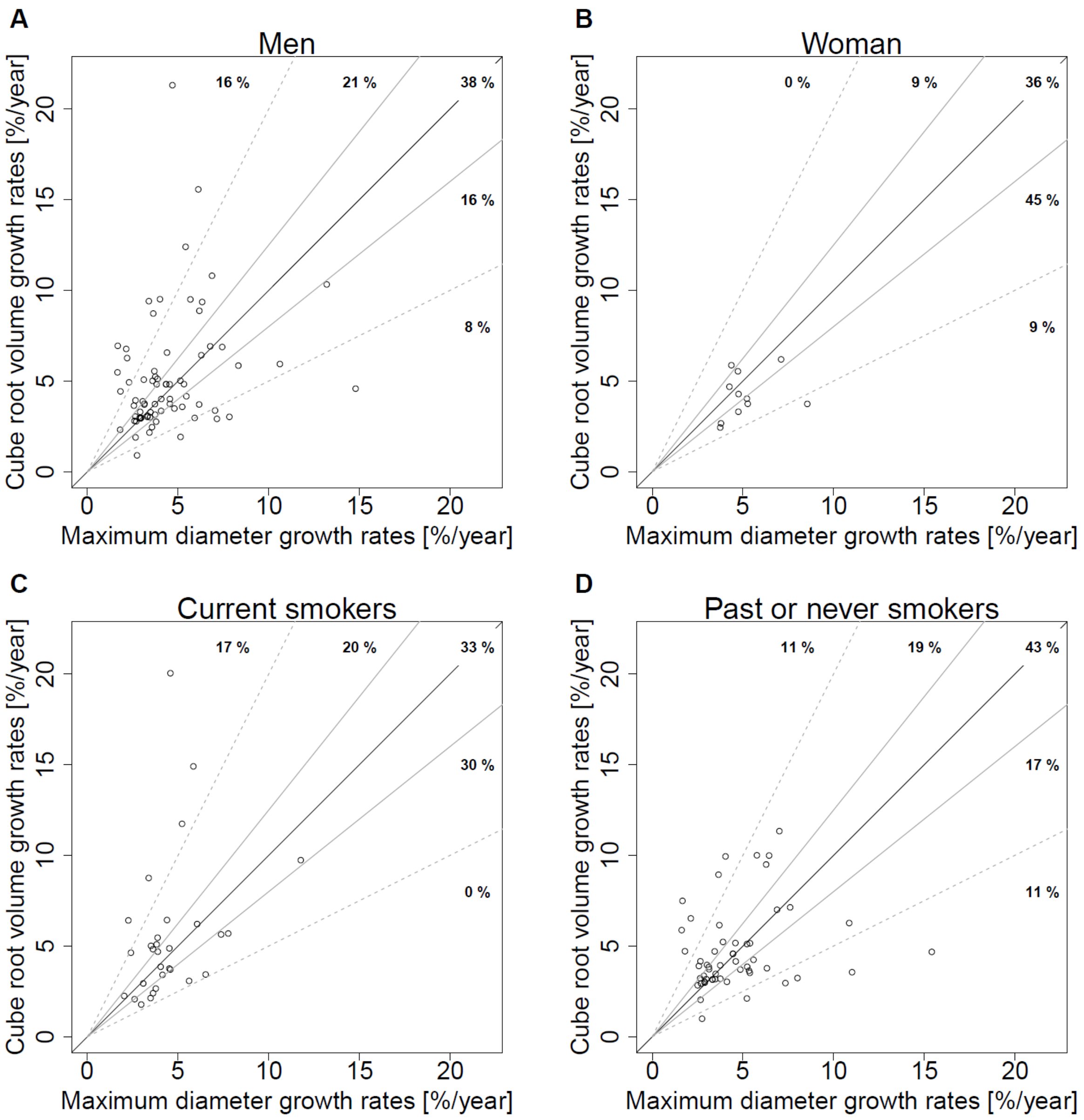

Supplementary Figure S1. Scatter plots of estimated individual growth rates for cube root volume and maximum diameter in the subgroups of men (A), women (B), current smokers (C) and past or never smokers (D). The diagonal solid black line indicates identical relative growth in both parameters. Solid grey lines represent ratios between the two growth rates of 0.8 and 1.25, dashed grey lines represent ratios of 0.5 and 2.0. Inlayed numbers show the proportion of patients within the respective reference ratios. Current smokers included 25 men and 5 women, past or never smokers included 46 men and 6 women.

Supplementary Table S2. Subgroup analysis of 1-year growth of AAA maximum diameter and volume. The investigated groups include the full sample (All) and the subgroups of men, women, current smokers and past or never smokers (labelled non-smokers). The table shows mean and median increase as well as selected quantiles of the growth distribution. The unit for all estimates is percent increase within one year. Standard errors are presented in parentheses below each value.

			**Quantiles of 1-year growth distribution**						
**Parameter**	**Subgroup**	**Mean**	**5%**	**10%**	**25%**	**Median**	**75%**	**90%**	**95%**
Diameter	All	5.2	0.5	1.2	2.3	4.1	6.8	10.4	13.3
		(0.4)	(0.2)	(0.2)	(0.2)	(0.3)	(0.5)	(0.9)	(1.3)
	Men	5.1	0.6	1.1	2.3	4.0	6.7	10.3	13.2
		(0.4)	(0.2)	(0.2)	(0.2)	(0.3)	(0.5)	(1.0)	(1.5)
	Woman	5.6	0.4	1.4	3.0	5.1	7.6	10.4	12.4
		(0.9)	(0.5)	(0.5)	(0.6)	(0.7)	(1.1)	(1.8)	(2.4)
	Smokers	4.9	0.4	1.1	2.4	4.2	6.6	9.6	12.0
		(0.6)	(0.3)	(0.3)	(0.3)	(0.4)	(0.7)	(1.3)	(1.9)
	Non-smokers	5.3	0.6	1.2	2.3	4.1	6.9	10.8	14.1
		(0.5)	(0.2)	(0.2)	(0.2)	(0.4)	(0.7)	(1.2)	(1.8)
Volume	All	19.3	-0.2	2.3	6.5	13.4	24.7	41.8	57.5
		(1.9)	(1.3)	(0.9)	(0.8)	(1.1)	(2.0)	(4.5)	(7.5)
	Men	20.2	-0.8	1.9	6.4	13.5	25.5	44.2	61.6
		(2.3)	(1.6)	(1.1)	(0.9)	(1.3)	(2.3)	(5.2)	(8.8)
	Woman	14.4	2.3	4.2	7.7	12.5	19.0	26.8	32.9
		(1.9)	(1.8)	(1.5)	(1.8)	(2.4)	(2.7)	(3.4)	(4.7)
	Smokers	(21.8	-2.2	1.1	6.1	14.0	27.4	48.7	69.1
		(4.2)	(3.1)	(1.9)	(1.5)	(2.0)	(4.1)	(9.3)	(15.5)
	Non-smokers	17.8	0.9	3.0	6.9	13.1	22.9	37.1	49.6
		(1.8)	(1.2)	(0.9)	(1.1)	(1.4)	(2.1)	(4.3)	(7.2)

3. Growth rates for volume and maximum diameter by aneurysm morphology

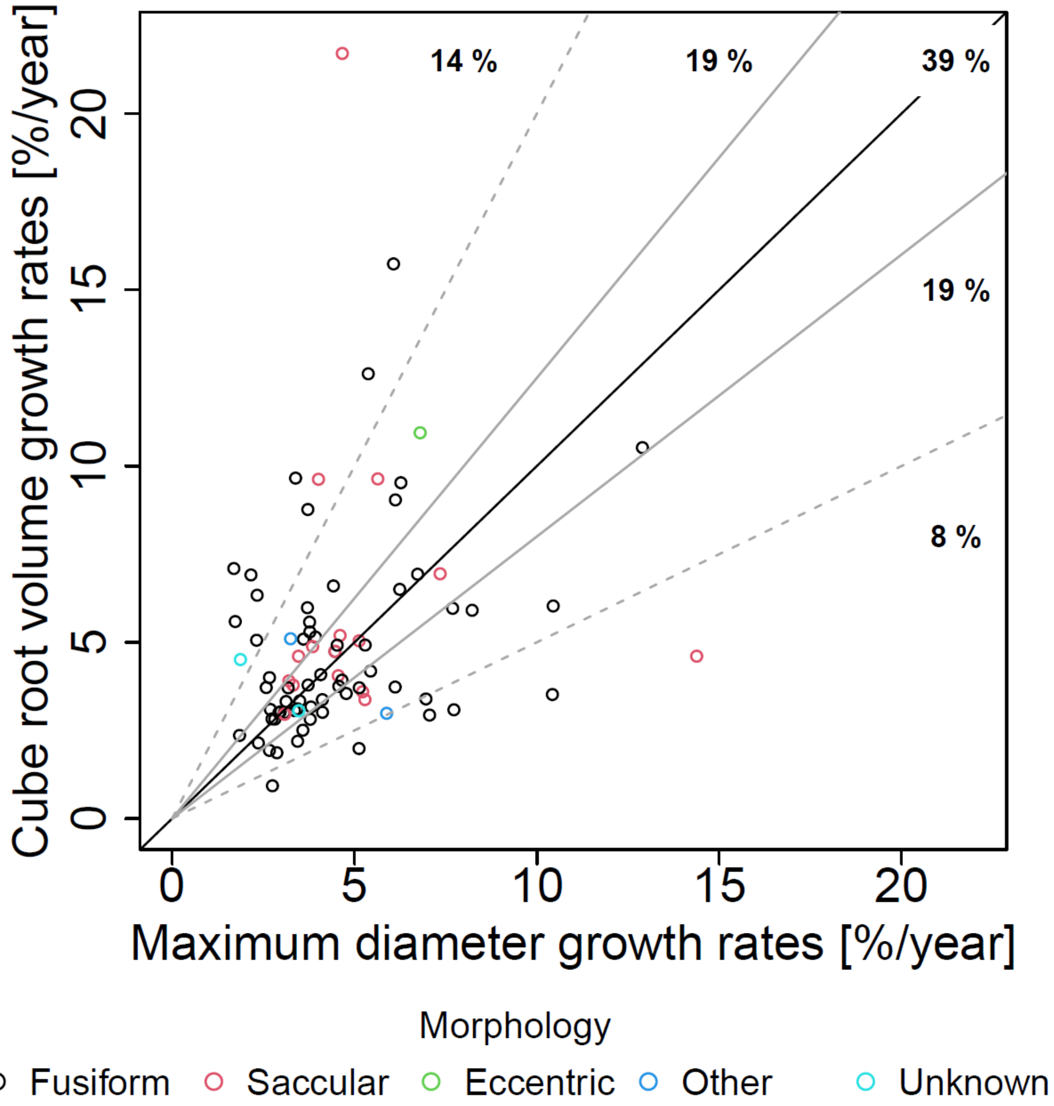

Supplementary Figure S2. Scatter plot of estimated individual growth rates for cube root volume and maximum diameter indicating different aneurysm morphology. The figure is based on Figure 3C of the manuscript, however, aneurysms of different morphological type are indicated by differently coloured symbols. The diagonal solid black line indicates identical relative growth in both parameters. Solid grey lines represent ratios between the two growth rates of 0.8 and 1.25, dashed grey lines represent ratios of 0.5 and 2.0. Inlayed numbers show the proportion of patients within the respective reference ratios.

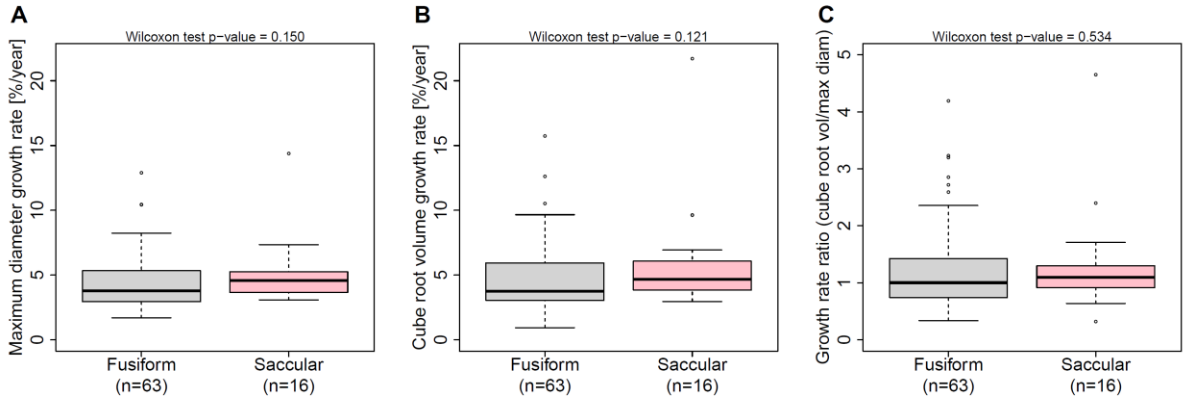

Supplementary Figure S3. Box plots of the distribution of estimated individual growth rates for (A) maximum diameter, (B) cube root volume and (C) their ratio, in fusiform and saccular aneurysms. Wilcoxon tests have been applied to compare the distribution of growth rates between fusiform and saccular aneurysms and the respective p-values are shown above each panel. In the subscripts, n refers to the numbers of observed fusiform and saccular aneurysms. The figure indicates that growth rates for maximum diameter (A) and for volume (B) are similar for fusiform and saccular aneurysms, with possible a small though not statistically significant difference in growth rates towards faster growth of saccular aneurysms. The heterogeneity in growth of volume versus growth of maximum diameter (C) is similar for fusiform and saccular aneurysms.
